# Supplementary material for: Chitosan–Silica Composites for Adsorption Application in the Treatment of Water and Wastewater from Anionic Dyes
Source: Int J Mol Sci. 2023 Jul 23;24(14):11818. doi: 10.3390/ijms241411818 (PMC10380244; doi:10.3390/ijms241411818)
Supplement: Supplementary file 1 [file ijms-24-11818-s001.zip › ijms-2508555-supplementary.pdf]

International Journal of *Molecular Sciences*

Supplementary Information

Chitosan–Silica Composites for Adsorption Application in the Treatment of Water and Wastewater from Anionic Dyes

Magdalena Blachnio [1,*], Malgorzata Zienkiewicz-Strzalka [1], Anna Derylo-Marczewska [1], Liudmyla V. Nosach [2] and Eugeny F. Voronin [2]

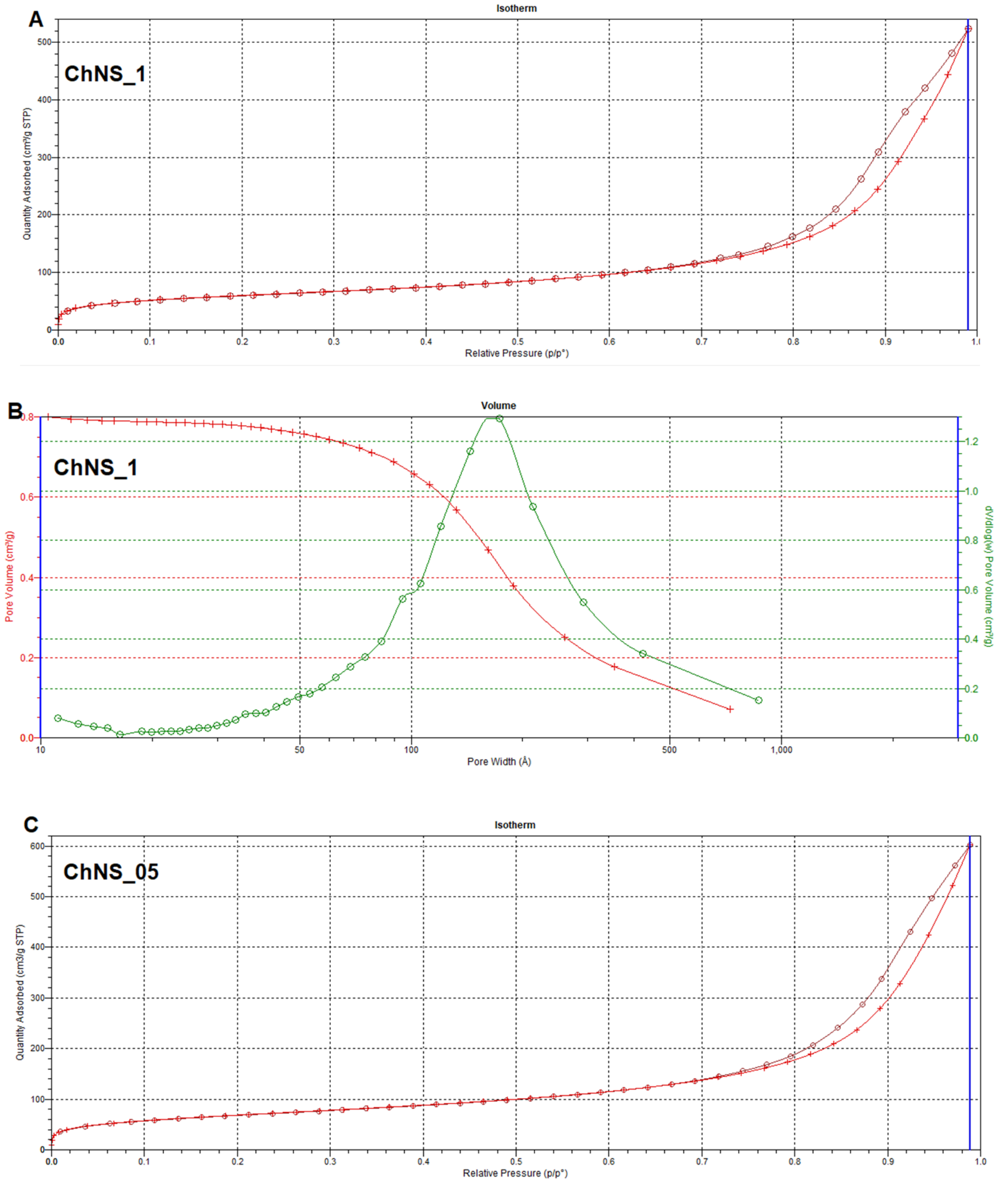

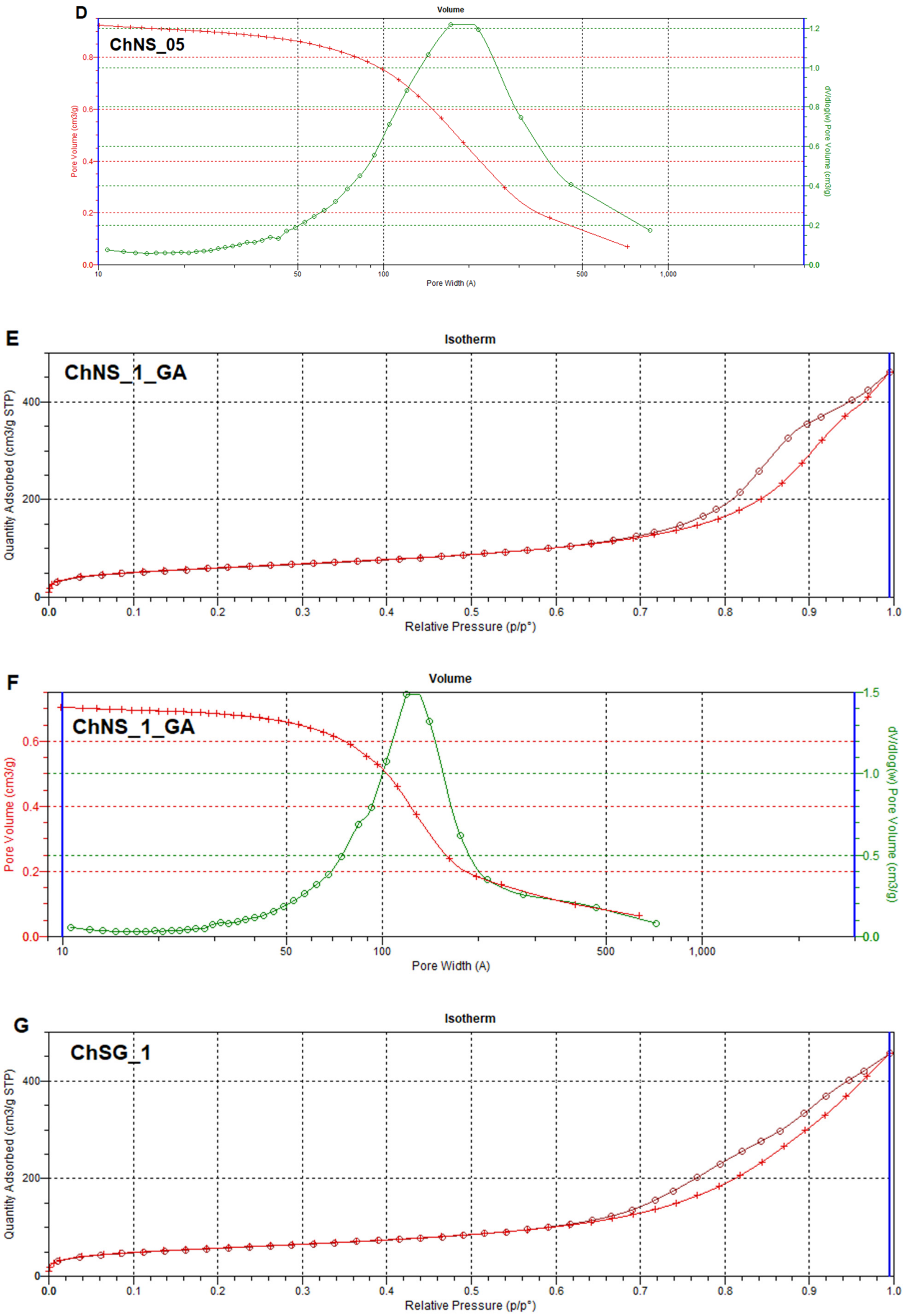
D
Volume
ChNS_05
Pore Volume (cm3/g)
dV/dlog(w) Pore Volume (cm3/g)
Pore Width (A)
E
Isotherm
ChNS_1_GA
Quantity Adsorbed (cm3/g STP)
Relative Pressure (p/p°)
F
Volume
ChNS_1_GA
Pore Volume (cm3/g)
dV/dlog(w) Pore Volume (cm3/g)
Pore Width (A)
G
Isotherm
ChSG_1
Quantity Adsorbed (cm3/g STP)
Relative Pressure (p/p°)

H

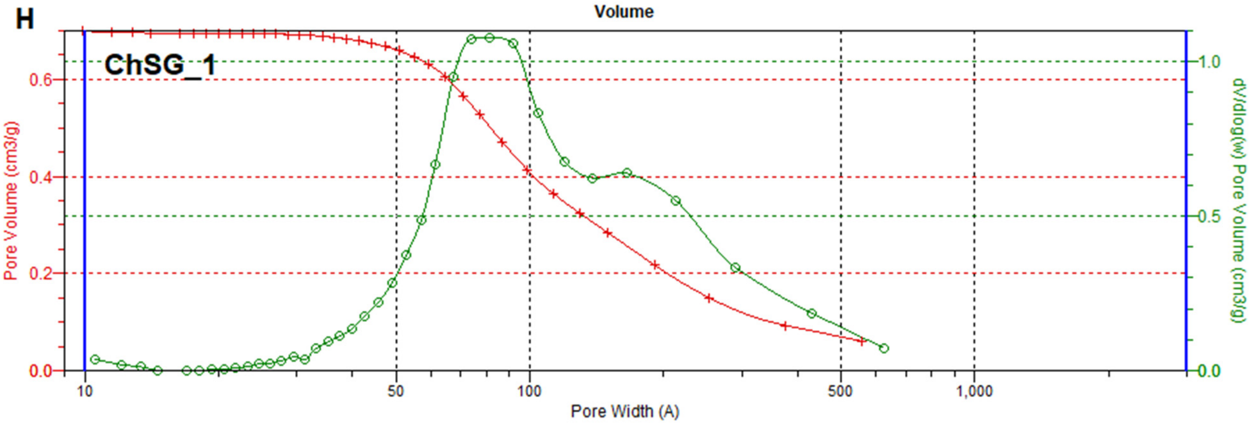
Volume
ChSG_1
Pore Volume (cm3/g)
dV/dlog(w) Pore Volume (cm3/g)
Pore Width (A)
0.0
0.2
0.4
0.6
0.0
0.5
1.0
10
50
100
500
1,000

I

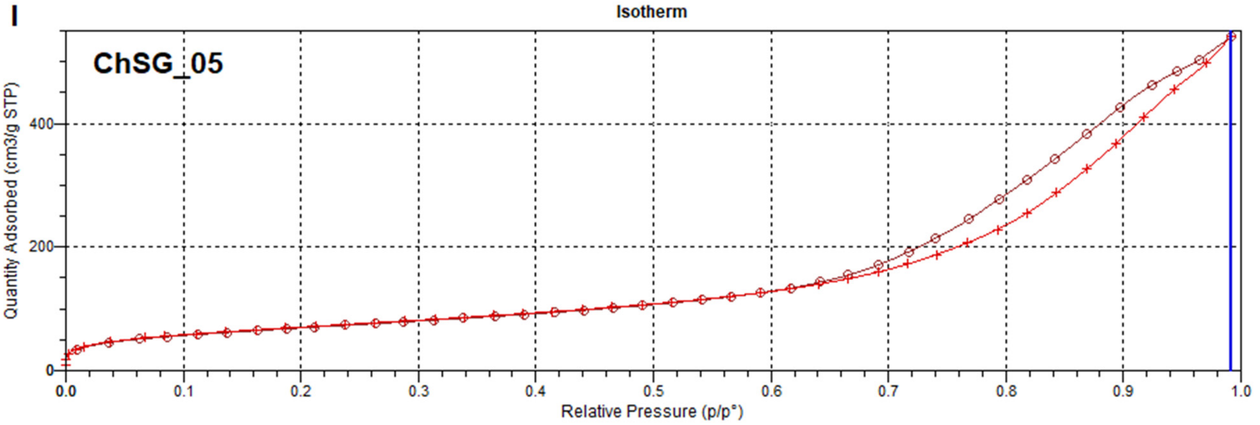
Isotherm
ChSG_05
Quantity Adsorbed (cm3/g STP)
Relative Pressure (p/p°)
0
200
400
0.0
0.1
0.2
0.3
0.4
0.5
0.6
0.7
0.8
0.9
1.0

J

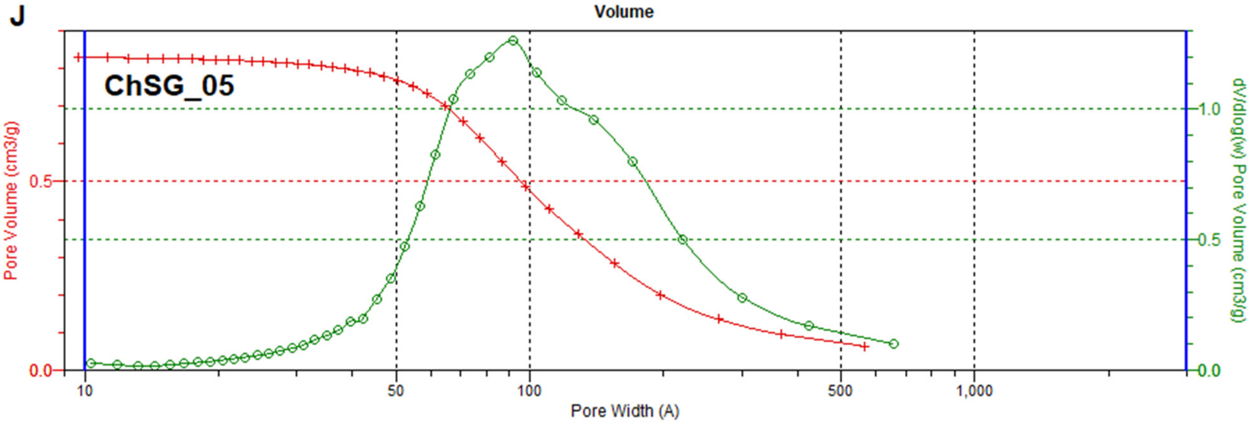
Volume
ChSG_05
Pore Volume (cm3/g)
dV/dlog(w) Pore Volume (cm3/g)
Pore Width (A)
0.0
0.5
0.0
0.5
1.0
10
50
100
500
1,000

K

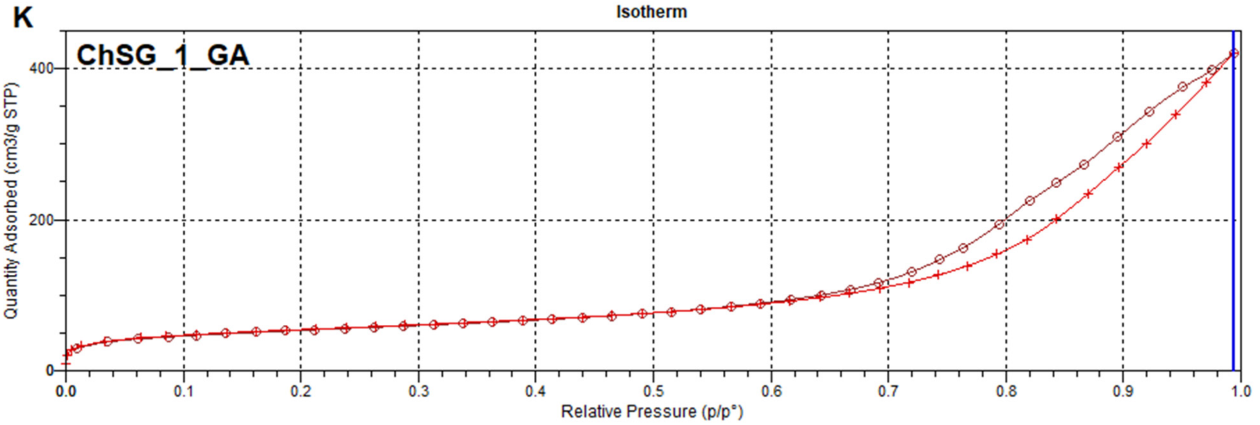
Isotherm
ChSG_1_GA
Quantity Adsorbed (cm3/g STP)
Relative Pressure (p/p°)
0
200
400
0.0
0.1
0.2
0.3
0.4
0.5
0.6
0.7
0.8
0.9
1.0

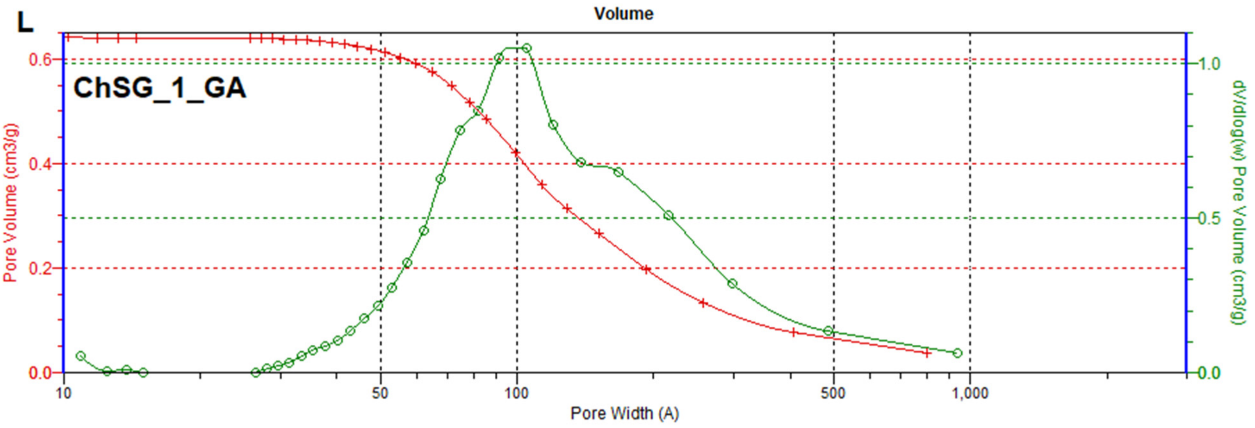

Figure S1. Low-temperature adsorption/desorption isotherms of nitrogen for all investigated samples and correlated BJH pore size distributions from desorption branch of isotherms.

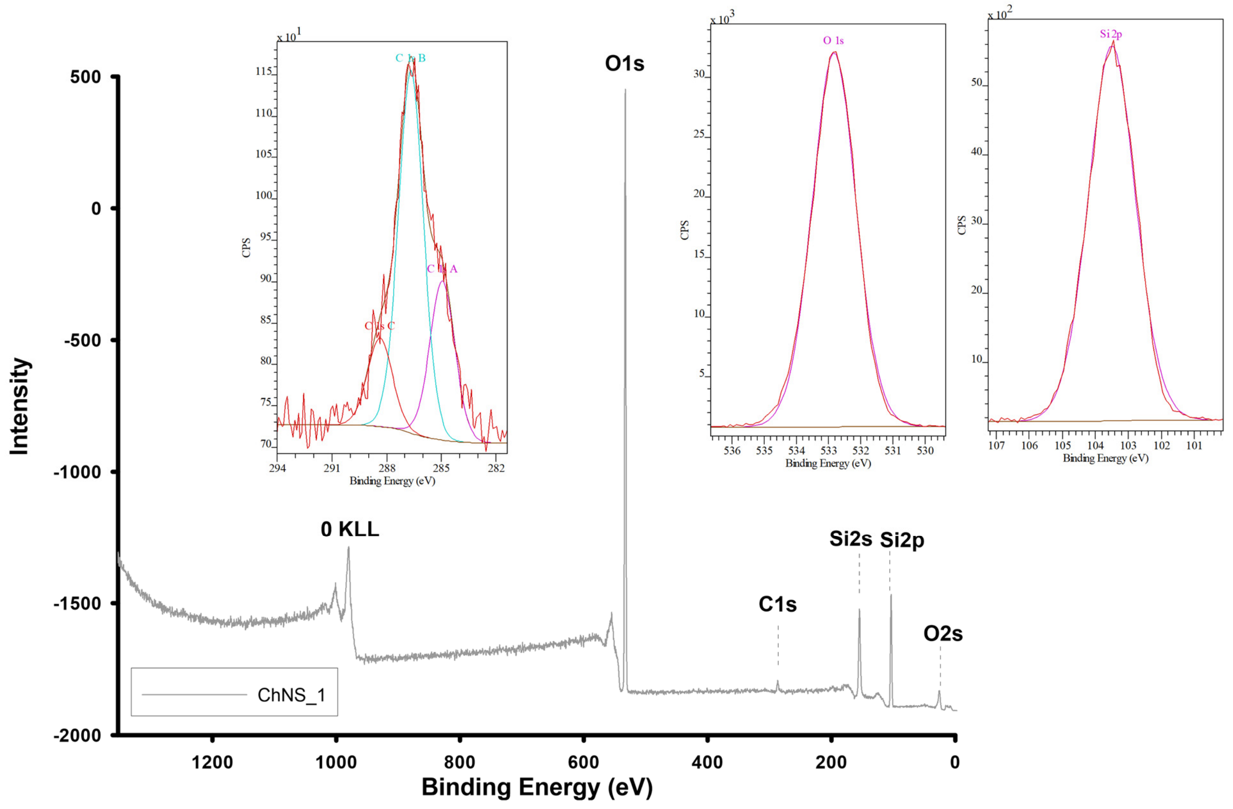

Name	%At Conc	% St.Dev.	%Mass Conc	Region	Name	Position	FWHM	Line Shape	Area	%At Conc	Type of interaction
Si2p	38.9	0.1	53.1	Si2p	Si2p	103.5	1.67	GL(30)	9851.4	100	SiO_2
O 1s	57.4	0.2	44.7	O 1s	O 1s	532.8	1.59	GL(30)	53999.8	100	SiO_2, C-O
C 1s	3.8	0.2	2.2	C 1s	C 1s A	284.9	1.6	GL(30)	335.2	26.1	C-C, C-H
					C 1s B	286.7	1.6	GL(30)	761.4	59.3	C-O
					C 1s C	288.4	1.6	GL(30)	186.4	14.5	O-C-O-

Figure S2. XPS analysis of selected chitosan-silica composite material (ChNS_1) and High-resolution XPS spectra of visible atoms Si2p, O1s and C1s.

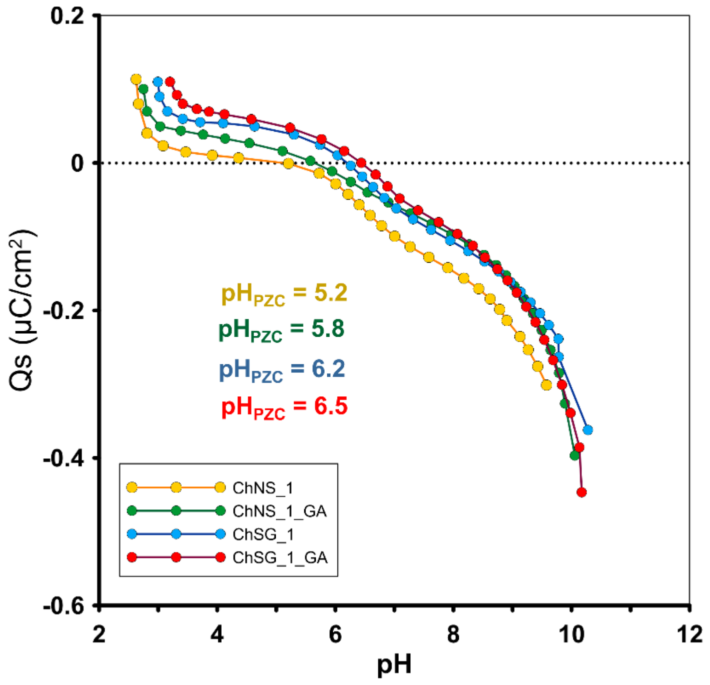

Figure S3. Surface charge density as a function of pH for selected samples ChNS_1, ChNS_1_GA, ChSG_1, and ChSG_1_GA.

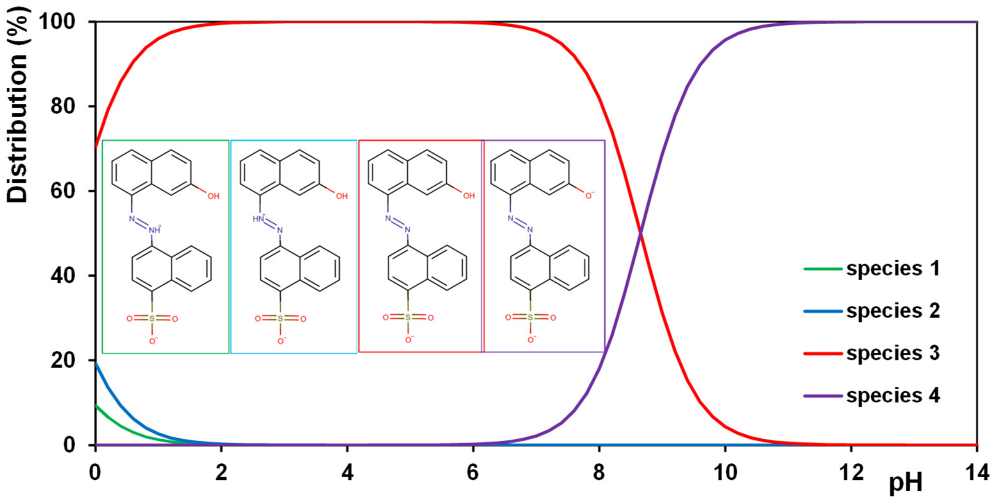

Figure S4. The percentage of the four molecular forms of the Acid Red 88 dye as a function of the solution pH.

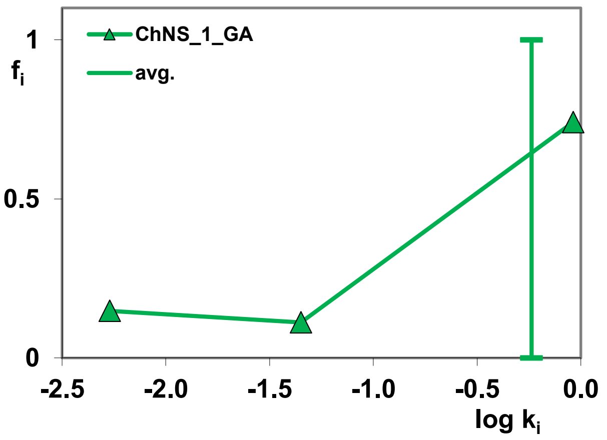

Figure S5. The distribution of adsorption rate coefficients k_i for AR 88 adsorption on the composite ChNS_1_GA.

In order to confirm the SiO_2 and chitosan interaction (covalent bonds) FTIR spectra were obtained for the initial chitosan and synthesized composite (ChNS_1). In the FTIR spectrum of chitosan the band at 3440 cm^{-1} corresponds to the stretching vibrations of hydroxyl groups O-H. Intensive absorption bands at 2880 to 2890 cm^{-1} are observed due to the C-H stretching vibrations. Position 1565 cm^{-1} corresponds to the deformation vibra-tions of -NH_2 and 1420 cm^{-1} for C-H bending vibrations. Asymmetric C-O-C stretching vibrations were found at 1300 cm^{-1} and 1070 cm^{-1} for C-O stretching vibration of CH-OH. In the case of the composite, we were looking for the vibration shift of the groups potentially involved in such bonding. These groups were the -NH_2 forms. The shift was observed in the range of 1565-1550 cm^{-1} and is shown in Figure S6. The content of chitosan in the composite was very low, therefore the intensity is relatively low, although observable.

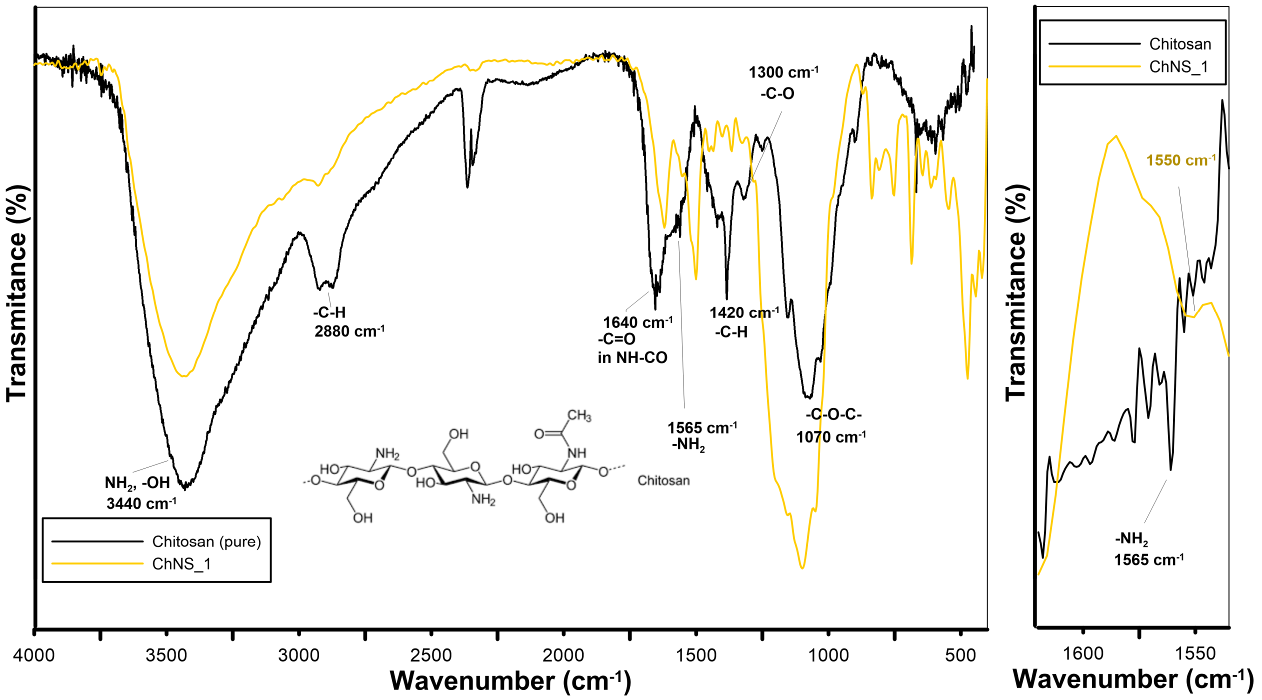

Figure S6. Structure characterization of chitosan and chitosan-silica nanocomposite (ChNS_1) by FTIR spectra. As insets: the chemical structure of chitosan and their functional groups correspond to the representative bands on the FTIR spectra and shift of position characteristic for –NH_2 groups as evidence of their interaction in composite system.

Table S1. The full parameters of the multi-exponential equation for exemplary system (dye/ChNS_1_GA).

Adsorbent	i	f_i	log k_i	$t_{0.5,i}$ [min]	log k_{avg}	$t_{0.5}$
ChNS_1_GA	1	0.74	-0.04	0.76	-0.24	1.20
	2	0.11	-1.35	15		
	3	0.15	-2.27	129		
